# Sirtuin 1 Regulates SREBP-1c Expression in a LXR-Dependent Manner in Skeletal Muscle

**DOI:** 10.1371/journal.pone.0043490

**Published:** 2012-09-11

**Authors:** Aurélia Defour, Kevin Dessalle, Andréa Castro Perez, Thomas Poyot, Josiane Castells, Yann Simon Gallot, Christine Durand, Vanessa Euthine, Yansong Gu, Daniel Béchet, André Peinnequin, Etienne Lefai, Damien Freyssenet

**Affiliations:** 1 Laboratoire de Physiologie de l'Exercice, Université de Lyon, Saint Etienne, France; 2 Laboratoire CarMeN, INSERM U1060, INRA 1235, Université de Lyon, Oullins, France; 3 Pôle de Génomique, Institut de Recherche Biomédicale des Armées, Centre de Recherche du Service de Santé des Armées, La Tronche, France; 4 Obenomics, Inc., Bellevue, Washington, United States of America; 5 INRA UMR 1019, Unité Nutrition Humaine, St Genès Champanelle, France; Université Joseph Fourier, France

## Abstract

Sirtuin 1 (SIRT1), a NAD^+^-dependent protein deacetylase, has emerged as a main determinant of whole body homeostasis in mammals by regulating a large spectrum of transcriptional regulators in metabolically relevant tissue such as liver, adipose tissue and skeletal muscle. Sterol regulatory element binding protein (SREBP)-1c is a transcription factor that controls the expression of genes related to fatty acid and triglyceride synthesis in tissues with high lipid synthesis rates such as adipose tissue and liver. Previous studies indicate that SIRT1 can regulate the expression and function of SREBP-1c in liver. In the present study, we determined whether SIRT1 regulates SREBP-1c expression in skeletal muscle. SREBP-1c mRNA and protein levels were decreased in the *gastrocnemius* muscle of mice harboring deletion of the catalytic domain of SIRT1 (SIRT1^Δex4/Δex4^ mice). By contrast, adenoviral expression of SIRT1 in human myotubes increased SREBP-1c mRNA and protein levels. Importantly, SREBP-1c promoter transactivation, which was significantly increased in response to SIRT1 overexpression by gene electrotransfer in skeletal muscle, was completely abolished when liver X receptor (LXR) response elements were deleted. Finally, our *in vivo* data from SIRT1^Δex4/Δex4^ mice and *in vitro* data from human myotubes overexpressing SIRT1 show that SIRT1 regulates LXR acetylation in skeletal muscle cells. This suggests a possible mechanism by which the regulation of SREBP-1c gene expression by SIRT1 may require the deacetylation of LXR transcription factors.

## Introduction

Protein acetylation is a tightly controlled process reciprocally regulated by histone acetyltransferases and histone deacetylases. The class III histone deacetylases also known as sirtuins (SIRTs) consists of seven members (SIRT1-7) [1], [2]. This is a highly conserved gene family encoding nicotinamide adenine dinucleotide (NAD)-dependent protein deacetylases and mono-ADP-ribosyl transferases [2]. SIRT1 is a member of the sirtuin family that deacetylates a variety of substrates and thus contributes to multiple cellular functions including stress responses, aging and cellular metabolism [3], [4]. The identification of a number of SIRT1 substrates in the last decade has clearly identified SIRT1 as a main determinant of whole-body metabolic homeostasis in several metabolically relevant tissues such as liver, adipose tissue and skeletal muscle [4]–[6]. SIRT1 deacetylates and regulates a large spectrum of transcriptional regulators, including p53 [7], [8], peroxisome-proliferated activated receptor γ coactivator (PGC)-1α [9], [10], forkhead-O-box transcription factors (FOXOs) [11], [12], liver X receptor (LXR) [13], and sterol regulatory element binding protein (SREBP)-1c [14]–[16].

SREBP proteins belong to the basic helix-loop-helix leucine zipper family of DNA binding transcription factors. SREBP-1a and -1c are produced from the same gene by alternative promoter usage, whereas a separate gene encodes SREBP-2 [17]. SREBP-1a and -1c control the expression of genes related to lipid metabolism, especially those involved in fatty acid and triglyceride synthesis, and SREBP-2 is related to the regulation of cholesterol synthesis [17]. Accordingly, SREBP transcription factors are markedly expressed in adipose tissue and liver, tissues with high rates of lipid synthesis [17]. However, SREBP-1a and -1c are also expressed to a significant level in skeletal muscle [18], [19], and more than 1,000 genes are regulated in response to SREBP-1a and SREBP-1c overexpression in primary human myotubes [20], suggesting additional functions of these transcription factors in skeletal muscle. Recent *in vivo* studies have suggested a crucial role for SIRT1 in regulating SREBP-1c expression and activity in liver. *In vivo*, SIRT1 activation decreased expression of SREBP-1c and SREBP-1c target genes [14]. Conversely, SIRT1 knockdown in liver induced the expression of SREBP-1c and its target genes encoding lipid-synthesizing enzymes [15]. Furthermore, SREBP-1c deacetylation by SIRT1 on Lys-289 and Lys-309 inhibits SREBP-1c activity by decreasing its stability and its association with lipogenic target genes [16], rendering SREBP-1c prone to ubiquitin-mediated degradation [21].

SREBP-1c expression is transcriptionally regulated by the nuclear receptors, liver X receptor (LXR) -α and -β, a family of transcription factors involved in the control of glucose, lipid and cholesterol metabolism [22]. LXRs can directly promote SREBP-1c transcription through two LXR response elements in the mouse SREBP-1c promoter [23]. Furthermore, synthetic LXR agonists up-regulate SREBP-1c gene expression both *in vivo* in rodents [24], and *in vitro* in cell models including human muscle cells [18], [25]. Interestingly, it has been reported that SIRT1 also deacetylates and activates the nuclear receptor LXR. In HEK293T cells, deacetylation at a single lysine residue of both LXR-α and LXR-β induces LXR activation, whereas SIRT1 deficiency in mice results in a decreased expression of LXR target genes in liver [13].

The above studies clearly show that SIRT1 can control SREBP-1c gene expression, at least in the liver, through a mechanism that may involve LXR transcription factors. However, it is currently unknown whether SIRT1 regulates SREBP-1c gene expression in skeletal muscle, a tissue with a low rate of lipid synthesis. We therefore addressed the question of whether SIRT1 regulates SREBP-1c gene expression in skeletal muscle by using a combination of animal studies, as well as molecular and cellular studies.

## Results

### SIRT1 regulates SREBP-1c expression in skeletal muscle cells

**Figure 1:**
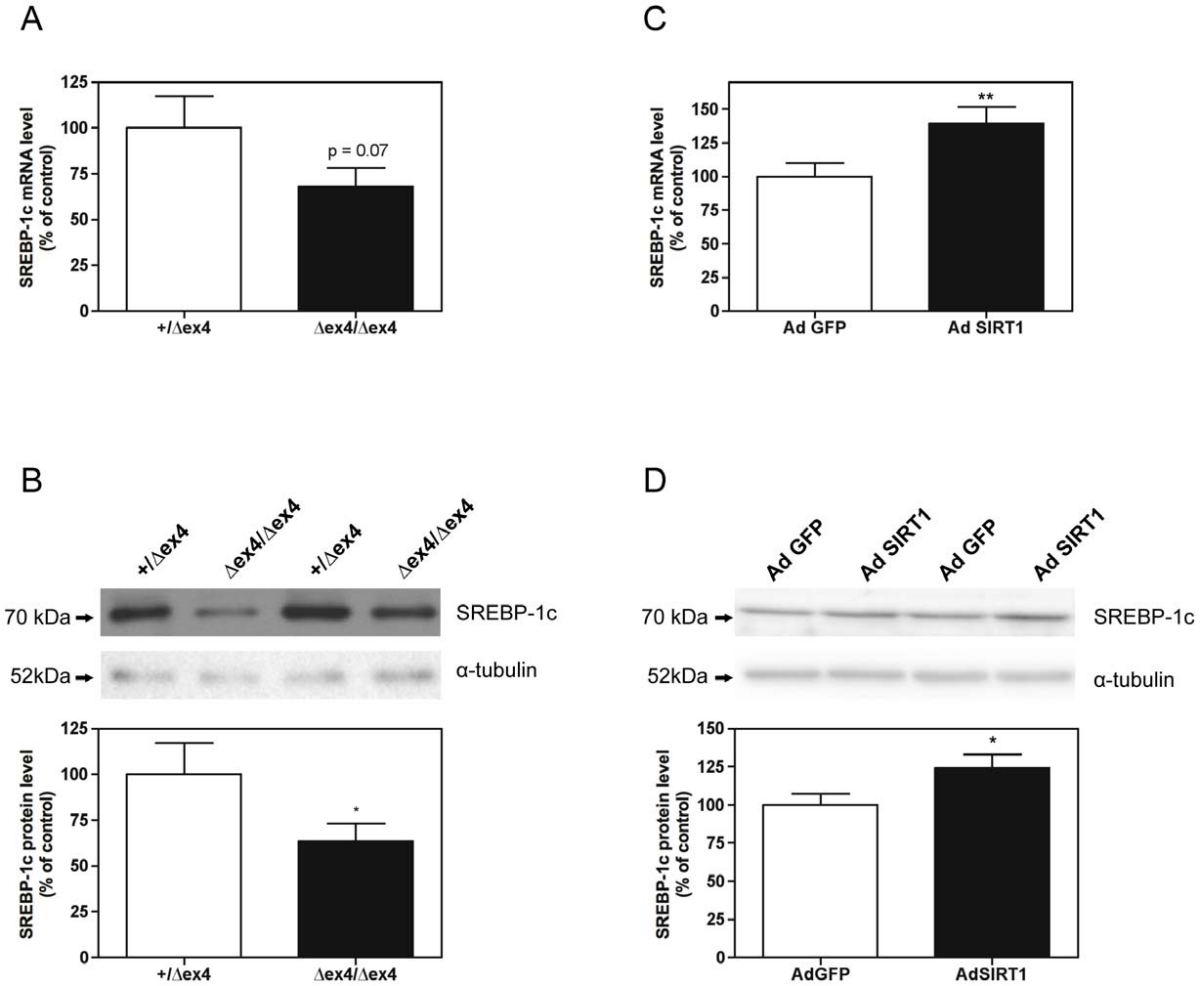
Sterol regulatory element binding protein (SREBP)-1c expression. (A) SREBP-1c mRNA level in the *gastrocnemius* muscle of SIRT1^+/Δex4^ and SIRT1^Δex4/Δex4^ mice. (B) SREBP-1c protein level in the *gastrocnemius* muscle of SIRT1^+/Δex4^ and SIRT1^Δex4/Δex4^ mice. (C) SREBP-1c mRNA level in human myotubes (7 days of differentiation) infected for 48 hours with recombinant adenovirus expressing either green fluorescent protein (AdGFP) or sirtuin 1 (AdSIRT1). (D) SREBP-1c protein level in human myotubes (7 days of differentiation) infected for 48 hours with AdGFP or AdSIRT1. For western blot analyses, equal protein loading was controlled by measuring total protein content and α-tubulin expression by western blot. Data are expressed as means ± SE (n = 4/group for animal study; n = 5/group for *in vitro* study). ** *P*<0.01 and * *P*<0.05: significantly different from the corresponding control condition.

**Figure 2:**
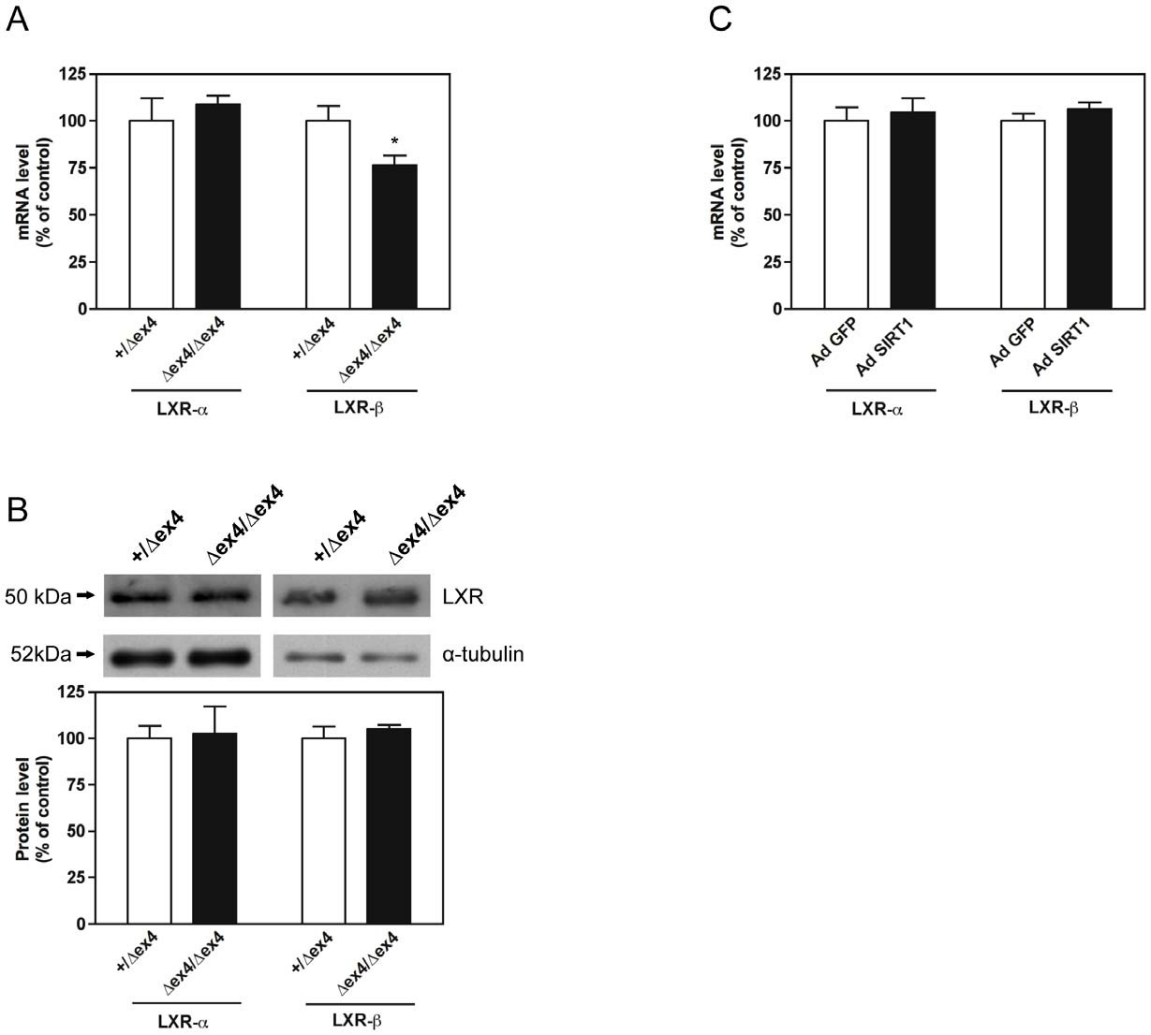
Liver X receptor (LXR)-α and LXR-β expression. (A) LXR-α and LXR-β mRNA level in the *gastrocnemius* muscle of SIRT1^+/Δex4^ and SIRT1^Δex4/Δex4^ mice. (B) LXR-α and LXR-β protein level in the *gastrocnemius* muscle of SIRT1^+/Δex4^ and SIRT1^Δex4/Δex4^ mice. (C) LXR-α and LXR-β mRNA level in human myotubes (7 days of differentiation) infected for 48 hours with recombinant adenovirus expressing either green fluorescent protein (AdGFP) or sirtuin 1 (AdSIRT1). For western blot analysis, equal protein loading was controlled by measuring total protein content and α-tubulin expression by western blot. Data are expressed as means ± SE (n = 4/group for animal study; n = 8/group for *in vitro* study). * *P*<0.05: significantly different from SIRT1^+/Δex4^ mice.

We first determined whether SIRT1 could control SREBP-1c expression in skeletal muscle. Both mRNA level and protein level of SREBP-1c were decreased in the *gastrocnemius* muscle of SIRT1^Δex4/Δex4^ mice compared to SIRT1^+/Δex4^ mice (Fig. 1*A–B*). Furthermore, adenoviral expression of SIRT1 in human myotubes significantly increased SREBP-1c mRNA and protein level (Fig. 1*C–D*). Taken together, these data indicate that SIRT1 can regulate SREBP-1c expression in skeletal muscle cells. Because LXR-α and LXR-β transcription factors are powerful inducers of SREBP-1c gene expression in skeletal muscle [18], we determined whether the reported decrease in SREBP-1c gene expression in SIRT1^Δex4/Δex4^ mice was associated by a concomitant decrease in LXR-α and LXR-β expression. Although the mRNA level of LXR-β was significantly decreased in the *gastrocnemius* muscle of SIRT1^Δex4/Δex4^ mice, LXR-α mRNA level, LXR-α protein level and LXR-β protein level remained unchanged in response to SIRT1 gene invalidation (Fig. 2 *A–B*). In agreement with these results, LXR-α and LXR-β mRNA level remained unchanged in response to adenoviral expression of SIRT1 in human myotubes (Fig. 2*C*). Therefore, SIRT1 does not appear to be a major regulator of LXR expression in skeletal muscle cells.

### SIRT1 regulates LXR-α and LXR-β acetylation in skeletal muscle cells

**Figure 3:**
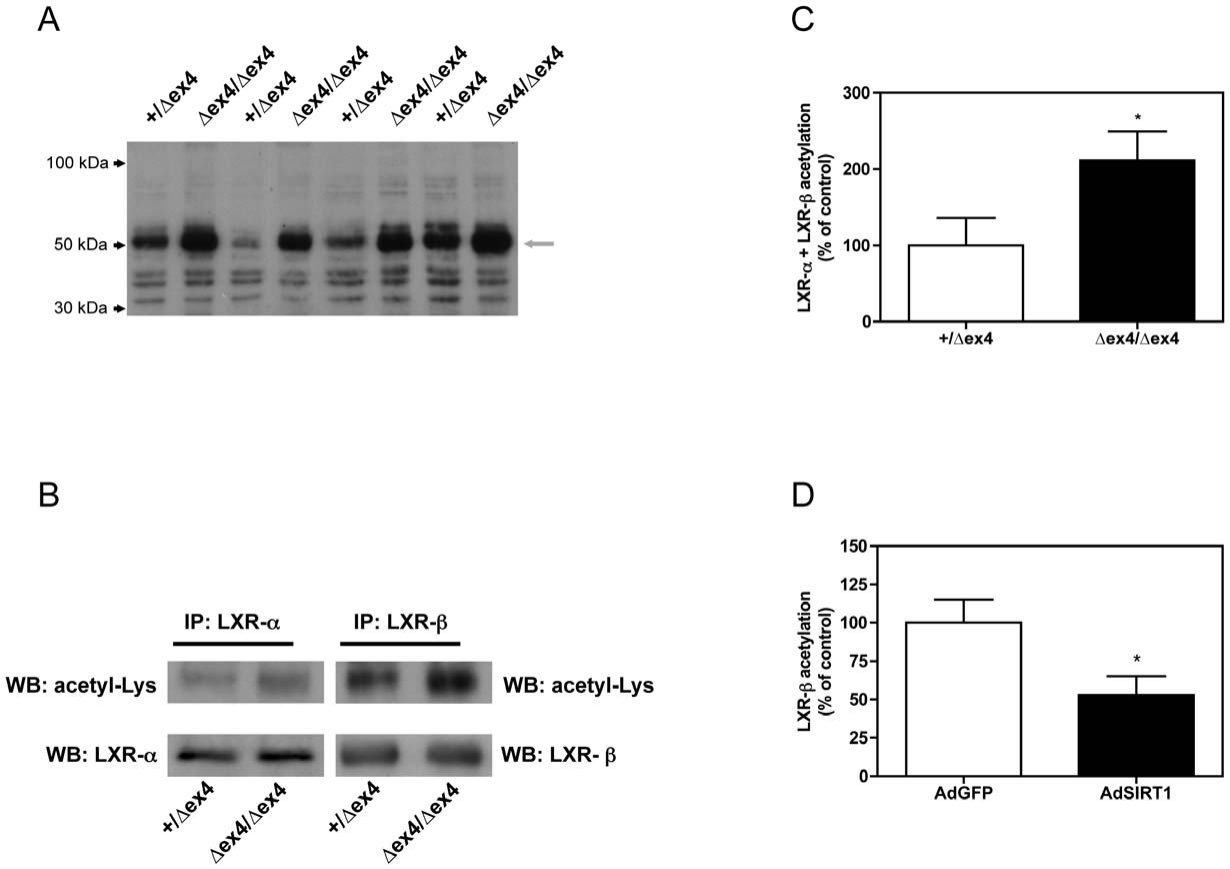
Sirtuin 1 (SIRT1) regulates liver X receptor (LXR)-α and LXR-β acetylation. (A) Immunoblot representing the acetylation profile of *gastrocnemius* muscle lysates of SIRT1^+/Δex4^ and SIRT1^Δex4/Δex4^ mice. Expected LXR band is indicated by a grey arrow. (B) Immunoblots showing LXR-α immunoprecipitates probed either with an acetyl-Lysine antibody (upper left) or a LXR-α antibody (lower left) and LXR-β immunoprecipitates probed either with an acetyl-Lysine antibody (upper right) or a LXR-β antibody (lower right). (C) Acetylation level of LXR-α and LXR-β normalized to the amount of LXR-α and LXR-β in the immunoprecipitate. (D) Acetylation level of LXR-β in human myotubes (7 days of differentiation) infected for 48 hours with recombinant adenovirus expressing either green fluorescent protein (AdGFP) or SIRT1 (AdSIRT1). Proteins were immunoprecipitated with a LXR-β antibody and then probed with an acetyl-lysine antibody. Data were normalized to the amount of LXR-β in the immunoprecipitate. Data are expressed as means ± SE (n = 4/group for animal study; n = 8/group for *in vitro* study). * *P*<0.05: significantly different from the corresponding control condition.

SIRT1 has been shown to deacetylate and increase LXR transcriptional activity in HEK293T cells [13]. We therefore reasoned that SIRT1 could control SREBP-1c gene expression by regulating LXR acetylation in skeletal muscle. We therefore determined the acetylation profile of the *gastrocnemius* muscle of SIRT1^+/Δex4^ and SIRT1^Δex4/Δex4^ mice. An hyperacetylated band at 50 kDa, which corresponds to the molecular weight of LXR isoforms, was particularly pronounced (Fig. 3*A*). We thus immunoprecipitated either LXR-α or LXR-β, and probed the resulting immunoprecipitate with an acetyl-lysine antibody. While the amount of LXR-α and LXR-β in the immunoprecipitates was fairly constant, acetylation of both LXR-α and LXR-β was significantly increased in the *gastrocnemius* muscle of SIRT1^Δex4/Δex4^ mice (Fig. 3*B–C*). Furthermore, adenoviral expression of SIRT1 significantly decreased LXR-β acetylation in human myotubes (Fig. 3*D*). LXR-α was modestly acetylated in control myotubes, and LXR-α acetylation was further decreased and below the detection limit in human myotubes overexpressing SIRT1 (data not shown). Altogether, these data show that SIRT1 regulates LXR-α and LXR-β acetylation level in skeletal muscle cells.

### SIRT1 overexpression regulates SREBP-1c promoter transactivation in a LXR-dependent manner *in vivo*

**Figure 4:**
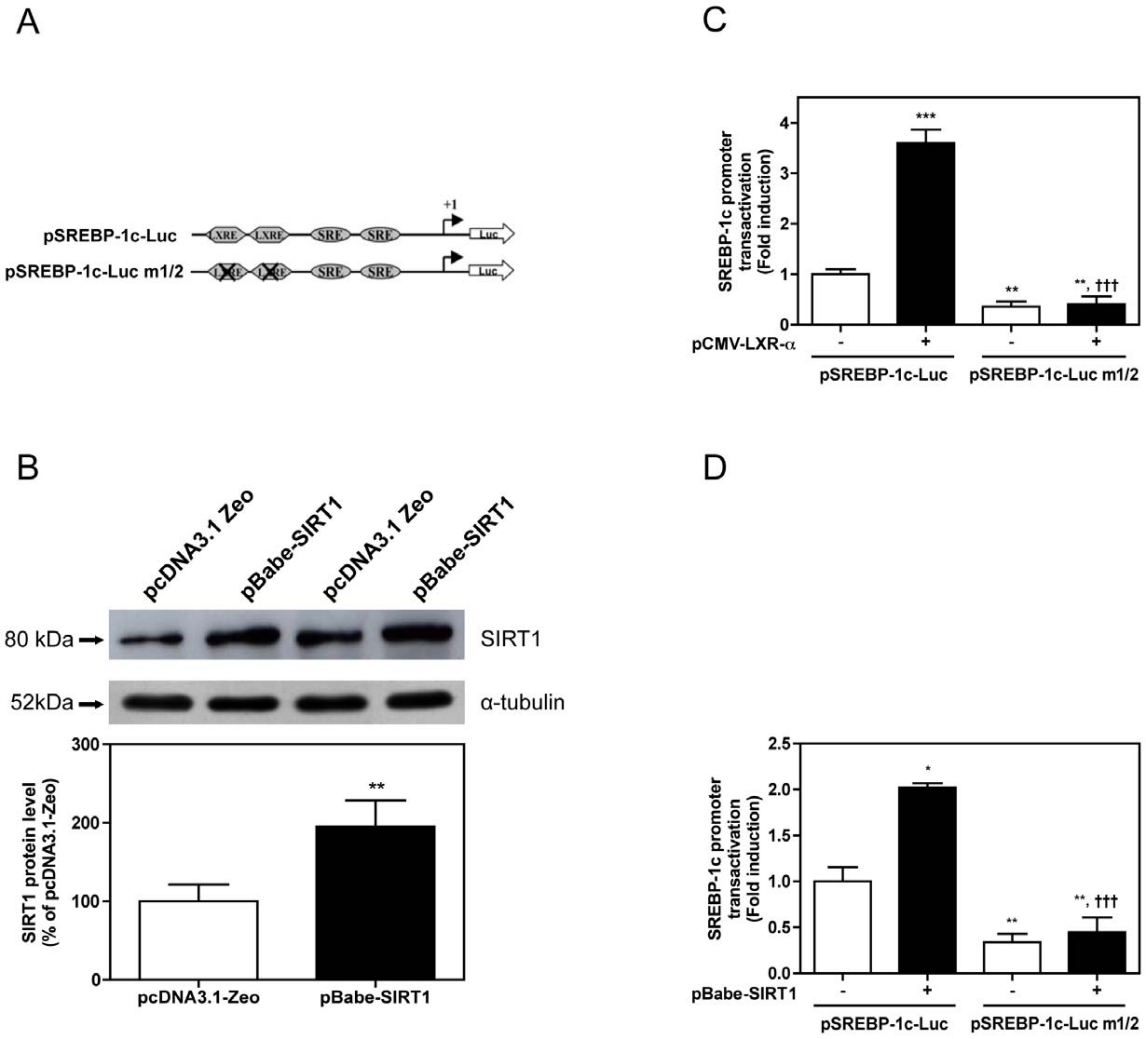
Sirtuin 1 (SIRT1) regulates sterol regulatory element binding protein (SREBP)-1c promoter transactivation. (A) Human SREBP-1c promoter (−571/+90 bp) reporter construct (pSREBP-1c-Luc) and human SREBP-1c promoter reporter construct with two deleted mutations in LXR-response elements located at −311/−296 bp and −260/−245 bp (pSREBP-1c-Luc m1/2) [18]. (B) SIRT1 protein level in mouse *tibialis anterior* muscle 7 days after electrotransfer of a control vector (pcDNA3.1-Zeo) or a SIRT1 expression vector (pBabe-SIRT1). Equal protein loading was controlled by measuring total protein content and α-tubulin expression by western blot. Data are means ± SE (n = 8/group). ** *P*<0.01: significantly different from pcDNA3.1-Zeo. (C) pSREBP-1c-Luc and pSREBP-1c-Luc m1/2 transactivation in absence and presence of a LXR-α expression vector (pCMV-LXR-α). *Tibialis anterior* muscles were removed 7 days after gene electrotransfer. Luciferase activity was normalized to β-galactosidase activity to correct for variations in transfection efficiency. Data are means ± SE (n = 8/group). ** *P*<0.01 and *** *P*<0.001: significantly different from pSREBP-1c-Luc alone. ††† *P*<0.001: significantly different from pSREBP-1c-Luc with pCMV-LXR-α. (D) pSREBP-1c-Luc and pSREBP-1c-Luc m1/2 transactivation in absence and presence of pBabe-SIRT1. *Tibialis anterior* muscles were removed 7 days after gene electrotransfer. Luciferase activity was normalized to β-galactosidase activity to correct for variations in transfection efficiency. Data are means ± SE (n = 8/group). * *P*<0.05 and ** *P*<0.01: significantly different from pSREBP-1c-Luc alone. ††† *P*<0.001: significantly different from pSREBP-1c-Luc with pBabeSIRT1.

To determine whether SIRT1 regulates SREBP-1c gene expression in a LXR-dependent manner, we next performed a gene electrotransfer experiment with SREBP-1c reporter gene constructs exhibiting or not mutations of two LXR-response elements [18] (Fig. 4*A*). We first checked that *in vivo* electrotransfer of a SIRT1 expression vector significantly increased SIRT1 protein content (Fig. 4*B*). As expected [18], the two SREBP-1c promoter constructs distinctly responded to LXR-α overexpression (Fig. 4*C*), indicating that the two LXR-binding sites are functional in our experimental setting and play an important role in the basal activity of the SREBP-1c promoter. As shown in Fig. 4*D*, SIRT1 overexpression significantly increased the transactivation of SREBP-1c promoter by about 2-fold, whereas SIRT1-induced transactivation of the SREBP-1c promoter was dramatically reduced when LXR motifs were mutated. These data demonstrate that the two LXR-response elements present in SREBP-1c promoter are involved in the stimulatory effect of SIRT1 on SREBP-1c promoter transactivation in mouse skeletal muscle.

## Discussion

Stable expression of the transcription factor SREBP-1c is essential for the regulation of lipid anabolism in tissues with high lipid synthesis rates such as liver and adipose tissue [17]. However, recent data indicate that SREBP-1c may also play important transcriptional regulatory functions in skeletal muscle [20], [26]. Here, we demonstrate that SIRT1 can regulate SREBP-1c expression in a LXR-dependent manner, that may involve LXR deacetylation. Taken together, these data provide novel insights into the regulatory mechanisms modulating SREBP-1c expression in skeletal muscle.

We showed that knocking out the catalytic domain of SIRT1 decreased SREBP-1c mRNA and protein levels, whereas adenoviral expression of SIRT1 increased SREBP-1c protein level. Furthermore, SREBP-1c promoter transactivation was significantly increased in response to SIRT1 overexpression by *in vivo* gene electrotransfer in skeletal muscle. Altogether, these data indicate that SIRT1 regulates SREBP-1c gene expression in skeletal muscle. Interestingly, recent data also indicate that SIRT1 mRNA and protein levels are increased in response to SREBP-1c adenoviral expression in human myotubes [20]. Together with our observations, this suggests the existence of a positive regulatory feedback loop: SIRT1 increases SREBP-1c expression, which then increases SIRT1 expression, thus ultimately further increasing SREBP-1c expression.

Previous studies indicate that LXR are powerful stimulators of SREBP-1c transcription both in liver [24] and skeletal muscle [18]. Here, our *in vivo* gene electrotransfer experiment showed that the transactivation of SREBP-1c promoter by SIRT1 was completely abolished when two functional LXR response elements were deleted in the SREBP-1c promoter. This indicates that SIRT1 regulates SREBP-1c gene expression in a LXR-dependent manner. Although the way by which SIRT1 regulates LXR transcriptional activity has not been directly explored in this study, this may involve a decrease in LXR acetylation. In support of this hypothesis, we show that LXR-α and LXR-β acetylation was significantly increased in the *gastrocnemius* muscle of SIRT1^Δex4/Δex4^ mice, and that adenoviral expression of SIRT1 decreased LXR-β acetylation in human myotubes. Furthermore, a previous report by Li *et al.* [13] showed that loss of SIRT1 reduced the expression of a variety of LXR target genes *in vivo*. They also showed that LXR-α and LXR-β are acetylated at Lys^432^ and Lys^433^ in HEK293T cells, and that SIRT1 promoted LXR deacetylation and subsequent ubiquitination and degradation [13]. Degradation of LXR paradoxically increases the recruitment of new LXR molecules at transcriptional active sites of target genes, ultimately leading to an increase in transcriptional activity [13]. Whether such a mechanism is functional in skeletal muscle remains to be determined.

Our data contrast with the negative regulatory role of SIRT1 on SREBP-1c expression and activity previously described in liver [14]–[16]. It has thus been reported that SIRT1 activation decreased SREBP-1c expression in the liver of genetically obese mice fed with a high-fat diet [14], and that knocking down SIRT1 induced SREBP-1c expression in the liver of fasted mice [15]. Importantly, these effects were all observed in response to metabolic challenges, and contrasts with the observation that SREBP-1c expression is increased in the liver of transgenic mice overexpressing SIRT1 in normal nutritional conditions [27]. Taken together, these data suggest that SIRT1 may have either negative or positive effects on SREBP-1c expression depending on metabolic context, tissue type and even promoter context.

One important question concerns the transcriptional targets of SREBP-1c regulated by SIRT1 in skeletal muscle. More than 500 genes have been shown to be either up- or down-regulated in response to SREBP-1c overexpression in human myotubes [20]. SIRT1 may thus exert multiple transcriptional regulatory functions in skeletal muscle via the control of SREBP-1c gene expression. As expected, SREBP-1c has been described to regulate the expression of genes involved in the control of lipid synthesis in skeletal muscle [19], [20], [28]. These data are in agreement with the reported increase in intramyocellular lipid content in skeletal muscle induced by resveratrol [29], a natural polyphenolic compound that indirectly activates SIRT1 through the activation of AMPK [30]–[32]. SREBP-1c has also been shown to regulate the expression of genes involved in the efflux of intracellular cholesterol to extracellular acceptors in C2C12 myotubes [28]. However, and although the action of SIRT1 on cholesterol metabolism has been well described in liver [13], [15], [33], [34], no effect of SIRT1 on cholesterol metabolism has been reported in skeletal muscle. Finally, SREBP-1c is also involved in the control of glucose metabolism in skeletal muscle. Previous studies thus indicate that one important transcriptional target of SREBP-1c in skeletal muscle is hexokinase II gene [19], [35], [36]. Furthermore, the expression of SIRT1 and hexokinase II both decreased in skeletal muscle of type II diabetic patients [37], [38]. One may therefore suggest that a defect in a SIRT1/LXR/SREBP-1c axis may contribute to alter the expression of hexokinase II. In agreement with this hypothesis, we observed that hexokinase II mRNA level and activity were both significantly decreased in the *gastrocnemius* muscle of SIRT1^Δex4/Δex4^ mice (Figure S1). However, further studies are necessary to evaluate the functional relevance of this observation in skeletal muscle.

In summary, we show that SIRT1 participates to the regulation of SREBP-1c expression in a LXR-dependent manner. We propose that this mechanism may involve the regulation of LXR acetylation.

## Materials and Methods

### Animals

Homozygous mice lacking exon 4 of the SIRT1 gene (SIRT1^Δex4/Δex4^ mice; 16.43±1.80 months; n = 4) and heterozygous mice lacking exon 4 of the SIRT1 gene (SIRT1^+/Δex4^ mice; 14.80±2.00 months; n = 4) were used [39]. Exon 4 encodes 51 amino acids of the conserved SIRT1 catalytic domain [39]. SIRT1^Δex4/Δex4^ mice thus expressed a SIRT1 mutant inactive protein, and are phenotypically identical to SIRT1 null mice [39]. SIRT1 heterozygous mice are phenotypically normal [39], [40] and were therefore used as controls. Morphological characteristics of SIRT1^Δex4/Δex4^ and SIRT1^+/Δex4^ mice are reported in Table S1. Mice were housed in a special pathogen-free facility. All procedures were approved by the University of Washington Animal Care and Use Committee.

Eight week-old male OF1 mice (32.9±1.2 g; n = 24) were used for gene electrotransfer experiments. The mice were maintained under a constant 12:12 h light-dark cycle with food and water *ad libitum*. All procedures were approved by Jean Monnet University Animal Care and Use Committee.

### Primary cell culture

Human muscle biopsies (∼200 mg wet weight) were taken under local anesthesia from the *vastus lateralis* muscle [25]. The experimental protocol was approved by the Ethical Committees of the Hospices Civils de Lyon and performed according to the French legislation. Written consent in accordance with the policy statement regarding the use of human subjects was obtained from all the subjects. Myoblasts were cultured in Ham's F10 supplemented with 2% Ultroser (BioSepra, Cergy-Saint-Christophe, France), 2% fetal bovine serum (FBS) and 1% penicillin/streptomycin (P/S) in 37°C and 5% CO_2_ in air. Human myoblasts were differentiated in Dulbecco's modified Eagle's medium supplemented with 2% FBS. Recombinant adenoviral genome carrying the human SIRT1 gene was generated by homologous recombination [38]. Five days differentiated human myotubes were infected for 2 days with adenovirus encoding SIRT1. Control myoblasts were infected with an adenovirus encoding the green fluorescent protein (GFP).

### Total RNA isolation and qRT-PCR

*Gastrocnemius* muscle samples were homogenized with a Mixer Mill MM300 (Retsch) in 300 µL Trizol (Invitrogen). Total RNA extraction was carried out on the Qiacube automate (Rneasy protocol). Total RNA from human myotubes was isolated by using the Trizol reagent following the manufacturer's instructions (Invitrogen). RT was immediately performed using the Reverse Transcription Core Kit (Eurogentec). The selected forward (FW) and reverse (RW) primer sequences are listed in Table S2. Real time PCR assay was performed in a 20 µl final volume and optimized concentration for each primer using a LightCycler Fast Start DNA Master SYBR Green kit (Roche Applied Science) and a LightCycler (Roche Applied Science). Relative quantification of samples was performed using the comparative threshold method [41], corrected for amplification efficiency variability between genes [42], and improved for use of multiple reference genes [43].

#### Immunoblot analysis

Muscle samples were homogenized (1∶20 dilution, wt/vol) at 4°C [50 mM Tris-HCl (pH = 7.4), 100 mM NaCl, 2 mM EDTA, 2 mM EGTA, 50 mM ß-glycerophosphate, 50 mM sodium fluoride, 1 mM Na_3_VO_4_, 120 nM okadaic acid, 1% Triton X-100]. Homogenates were centrifuged at 12,000 g for 20 min at 4°C. Human myotube lysates were prepared as previously described [18]. Protein concentration was spectrophotometrically measured at 750 nm using the Bio-Rad *DC* protein assay. Depending on the antibody used, either 20 or 50 µg of proteins were subjected to SDS-PAGE and transferred to nitrocellulose membranes. The blots were incubated overnight at 4°C with antibodies against LXR-α (1∶400, sc-1202, Santa Cruz Biotechnology), LXR-β (1∶400, sc-1001, Santa Cruz Biotechnology), SIRT1 (1∶1,000, Upstate 05707) and nuclear SREBP-1c (1∶40, Santa Cruz Biotechnology sc-8984). Corresponding horseradish peroxidase-conjugated rabbit anti-mouse (1∶3,000, Dako) and goat anti-rabbit (1∶3,000, Dako) antibodies were used for chemiluminescent protein detection (ECL, GE Health Care). Equal protein loading was controlled by measuring α-tubulin content by western blot. Membranes were stripped (Re-Blot Plus Strong Solution, Millipore), checked for the absence of immunoreactivity, incubated with α-tubulin (1∶500, Sigma T5168), and then processed as described above. The films were scanned and quantified using ImageJ analysis.

### Immunoprecipitation

Protein extracts (200 µg) from *gastrocnemius* skeletal muscle or human myotubes were incubated for one hour at 4°C with 2 µg of LXR antibodies (LXR-α sc-1202, LXR-β sc-1001, Santa Cruz Biotechnology) in 1 ml of RIPA buffer [50 mM Tris-HCl (pH = 7.4), 150 mM NaCl, 1 mM EDTA, 1 mM MgCl_2_, 1% NP40]. Protein magnetic B sepharose beads (Millipore) were then added and incubated at 4°C for an additional 15 minutes. The beads were washed 3 times with RIPA buffer and finally suspended in Laemmli's sample buffer. Proteins were resolved on 10% SDS-PAGE gel and immunoblotted using an acetyl-lysine antibody (1∶500, Ac-K-103, #9681, Cell Signaling). The membranes were stripped (Re-Blot Plus Strong Solution, Millipore), checked for the absence of immunoreactivity, and then reprobed with the corresponding LXR antibody.

### Plasmids

pBabe-SIRT1, the full-length murine SIRT1 cDNA in a pBabepuro expression vector, was a gift from Dr J Luo (Columbia University, New York, USA) [7]. pCMV-LXR-α (Source BioScience/Geneservice, Cambridge, UK) is the full length murine LXR-α cDNA in a pCMV-Sport vector. Human SREBP-1c promoter (−571/+90 bp) reporter gene (pSREBP-1c-Luc) and human SREBP-1c promoter (−571/+90 bp) reporter gene with two deleted mutations in LXR-response elements located at −311/−296 bp and −260/−245 bp (pSREBP-1c-Luc m1/2) were previously described [18] (Fig. 4*A*). pCMV-ß-galactosidase was obtained from Clontech. pcDNA3.1-Zeo (Clontech) is a null empty vector used to maintain constant the amount of DNA injected.

### *In vivo* gene electrotransfer

Mice were anesthetized (*i.p.* injection of 90 mg/kg ketamine and 10 mg/kg xylazine) and *tibialis anterior* muscles were injected with 125 µl of DNA mixtures in 0.9% NaCl endotoxin-free containing: *i)* 30 µg of pSREBP-1c-Luc, 30 µg of pCMV-ß-galactosidase, and 20 µg of pcDNA3.1-Zeo (n = 8), *ii)* 30 µg of pSREBP-1c-Luc, 30 µg of pCMV-ß-galactosidase and 20 µg of pBabe-SIRT1 (n = 8), and *iii)* 30 µg of pSREBP-1c-Luc, 30 µg of pCMV-ß-galactosidase and 20 µg of pCMV-LXR-α (n = 8). Corresponding contralateral muscles were injected with the same DNA mixture except that pSREBP-1c-Luc was omitted and replaced by 30 µg of pSREBP-1c-Luc m1/2. Thirty seconds after injection, 6 pulses (20 ms, 50 mA) were delivered using a GET42 electroporator as previously described [44]–[46].

### Firefly luciferase and ß-galactosidase assays

Seven days after gene electrotransfer, the animals were anesthetized and the *tibialis anterior* muscles were removed. Protein isolation and measurement of firefly luciferase activity were performed as described previously [45]. To correct for interindividual variations in transfection efficiency, luciferase activity was normalized to β-galactosidase activity [45].

### Statistics

Data are means ± SE. Statistical comparisons between SIRT1^Δex4/Δex4^ and SIRT1^+/Δex4^ mice, as well as statistical comparisons between AdGFP and AdSIRT1 myotubes were performed using unpaired t-test. The effect of SIRT1 gene electrotransfer on SIRT1 protein level was determined by using a paired t-test. A two-way analysis of variance was used to determine the effects of LXR-α and SIRT1 expression on the transactivation of pSREBP-1c-Luc and pSREBP-1c-Luc m1/2 promoters. Fisher's post hoc test was then used to determine specific mean differences. All statistical analyses were performed using StatView (StatView™SE+^Graphics^, Abacus Concept, Inc). The 0.05 level of confidence was accepted as the threshold for statistical significance.

## Supporting Information

Figure S1 **Hexokinase (HK) activity in the** ***tibialis anterior*** **muscle of Sirt1^+/Δex4^ and Sirt1^Δex4/Δex4^ mice.** HK activity was determined by fluorimetric analysis. Data are expressed as means ± SE (n = 4/group). ** *P*<0.01: significantly different from SIRT1^+/Δex4^ mice.
(TIF)

Table S1 **Morphometric characteristics of Sirt1^+/Δex4^ and Sirt1^Δex4/Δex4^ mice.**
(DOCX)

Table S2 **Primers sequences and annealing temperatures of genes used for qPCR analysis.**
(DOCX)
