# Supplementary figures and images for: Sirtuin 1 Regulates SREBP-1c Expression in a LXR-Dependent Manner in Skeletal Muscle
Source: PLoS One. 2012 Sep 11;7(9):e43490. doi: 10.1371/journal.pone.0043490 (PMC3439460; doi:10.1371/journal.pone.0043490)

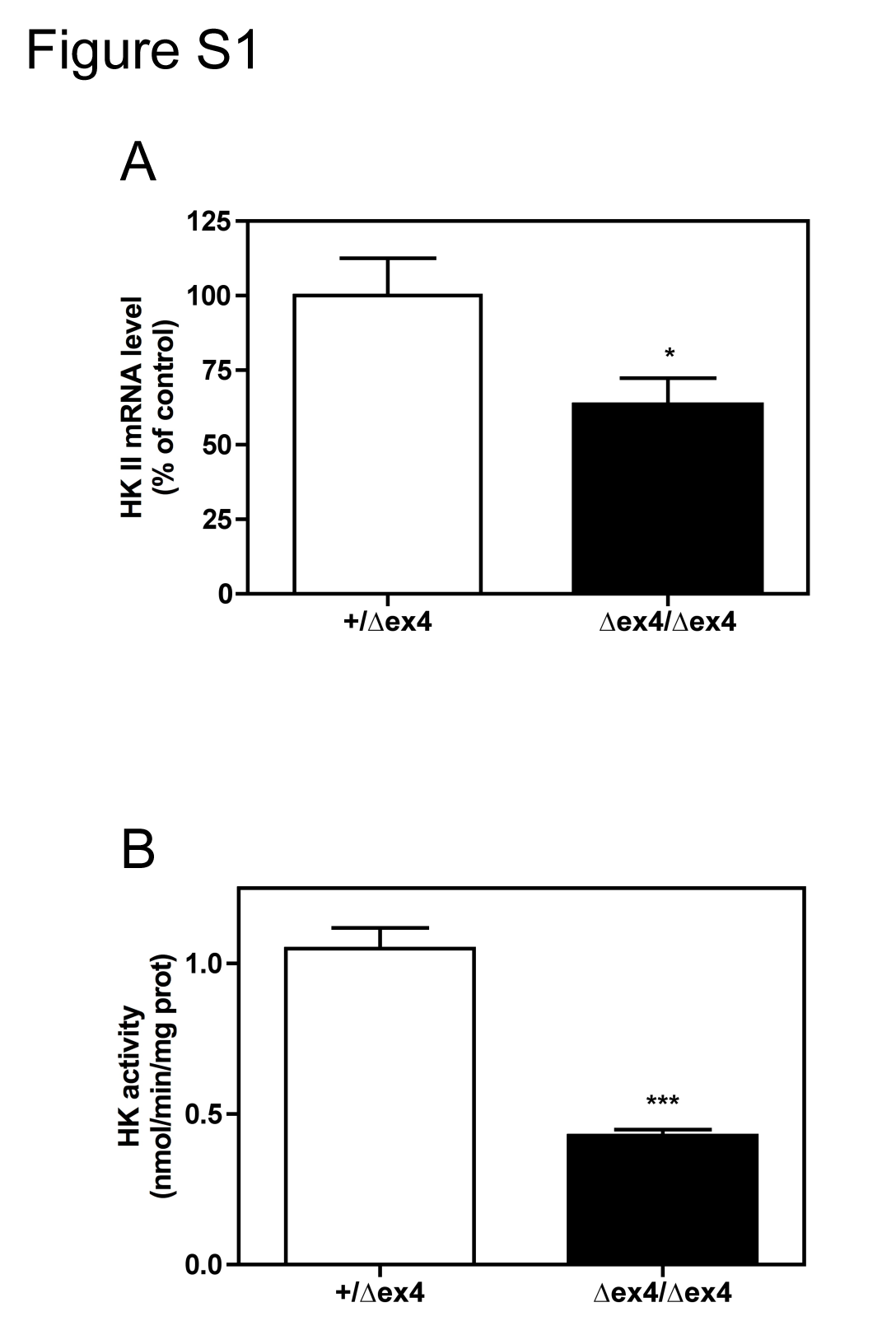

Supplement: Figure S1 — Hexokinase (HK) activity in the tibialis anterior muscle of Sirt1+/Δex4 and Sirt1Δex4/Δex4 mice. HK activity was determined by fluorimetric analysis. Data are expressed as means ± SE (n = 4/group). ** P<0.01: significantly different from SIRT1+/Δex4 mice. (TIF) [file pone.0043490.s001.tif]
