# Supplementary material for: Sirtuin 1 Regulates SREBP-1c Expression in a LXR-Dependent Manner in Skeletal Muscle
Source: PLoS One. 2012 Sep 11;7(9):e43490. doi: 10.1371/journal.pone.0043490 (PMC3439460; doi:10.1371/journal.pone.0043490)
Supplement: Table S1 — Morphometric characteristics of Sirt1+/Δex4 and Sirt1Δex4/Δex4 mice. (DOCX) [file pone.0043490.s002.docx]

Supplementary Table 1. **Morphometric characteristics of Sirt1^+/Δex4^ and Sirt1^Δex4/Δex4^ mice**

| Characteristics | Sirt1^+/∆ex4^ | Sirt1^∆ex4/∆ex4^ |
| --- | --- | --- |
| Body weight (g) | 33.78 ± 1.86 | 22.80 ± 1.15 ** |
| Body mass index | 30.92 ± 1.52 | 27.03 ± 1.52 |
| *Gastrocnemius* weight (mg) | 137.00 ± 12.00 | 92.00 ± 8.00 * |
| *Tibialis* *anterior* weight (mg) | 50.00 ± 5.00 | 35.00 ± 3.00 * |
| *Extensor digitorum longus* weight (mg) | 10.40 ± 0.12 | 7.93 ± 0.62 * |
| *Soleus* weight (mg) | 5.89 ± 0.67 | 4.20 ± 0.45 ^p = 0.08^ |
| *Gastrocnemius* (mg/g of body weight) | 4.06 ± 0.33 | 4.01 ± 0.24 |
| *Tibialis anterior* (mg/g of body weight) | 1.49 ± 0.15 | 1.52 ± 0.12 |
| *Extensor digitorum longus* (mg/g of body weight) | 0.29 ± 0.02 | 0.31 ± 0.03 |
| *Soleus* (mg/g of body weight) | 0.18 ± 0.02 | 0.19 ± 0.01 |

Data are expressed as means ± SE (n = 4/group). **P* < 0.05 and ***P* < 0.01: significantly different from Sirt1^+/Δex4^ mice.
