# Supplementary material for: Sirtuin 1 Regulates SREBP-1c Expression in a LXR-Dependent Manner in Skeletal Muscle
Source: PLoS One. 2012 Sep 11;7(9):e43490. doi: 10.1371/journal.pone.0043490 (PMC3439460; doi:10.1371/journal.pone.0043490)
Supplement: Table S2 — Primers sequences and annealing temperatures of genes used for qPCR analysis. (DOCX) [file pone.0043490.s003.docx]

Supplementary Table 2. **Primers sequences and annealing temperatures of genes used for qPCR analysis**

| Gene | GenBank Accession no. | 5’-3’ sequence of forward (FW) and reverse (RW) primers | Annealing temperature (°C) |
| --- | --- | --- | --- |
| ARBP | [NM_007475](http://www.ncbi.nlm.nih.gov/nuccore/NM_007475.5) | FW GAAAATCTCCAGAGGCACCATTG  RW TCCCACCTTGTCTCCAGTCTTTATC | 54 |
| CYCA | [NM_008907](http://www.ncbi.nlm.nih.gov/nuccore/NM_013556.2) | FW GGCAAATGCTGGACCAAACAC  RW CTTCCCAAAGACCACATGCTTG | 54 |
| HK II | NM_013820 | FW CAACCCTGGCAAACAGAGGTT  RW AGCCCCCGCTTTGTGAAAT | 56 |
| HPRT | [NM_013556](http://www.ncbi.nlm.nih.gov/nuccore/NM_013556.2) | FW CTCATGGACTGATTATGGACAGGAC  RW GCAGGTCAGCAAAGAACTTATAGCC | 60 |
| LXR-α | NM_013839 | FW TCCAAGTAGAGAGGCTGCAACAC  RW TTAGCATCCGTGGGAACATCAG | 57 |
| LXR-α | NM_005693 | FW CCGGGAAGACTTTGCCAAAGC  RW GGAGCTGGTCCTGCACGTTG |  |
| LXR-β | NM_009473 | FW GAAGGCGTCCACCATTGAGAT  RW AGTCGTCCTTGCTGTAGGTGAAGT | 56 |
| LXR-β | NM_007121 | FW AGGACCAGATCGCCCTCCTG  RW GGTGGAAGTCGTCCTTGCTGTAGG |  |
| SREBP-1c | NM_011480 | FW CGGGATGCGGCTGTTGTCT  RW GCAGCAAGATGTCCTCCTGTGTACT | 56 |
| SREBP-1c | N_M001005291 | FW GCAGATCGCGGAGCCATGGATTGC  RW GAGGTGGAGACAAGCTGCCTGG | 60 |
| TBP | N_M003194 | FW TGGTGTGCACAGGAGCCAAG  RW TTCACATCACAGCTCCCCAC | 60 |
|  |  |  |  |

ARBP: acidic ribosomal phosphoprotein; CYCA: cyclophilin A; HK II: hexokinase II; HPRT: hypoxanthine guanine phosphoribosyl transferase; LXR: liver X receptor; SREBP: sterol regulatory element binding protein; TBP: TATA box binding protein.
